# Administration of anti-inflammatory M2 macrophages suppresses progression of angiotensin II-induced aortic aneurysm in mice

**DOI:** 10.1038/s41598-023-27412-x

**Published:** 2023-01-25

**Authors:** Shinichi Ashida, Aika Yamawaki-Ogata, Masayoshi Tokoro, Masato Mutsuga, Akihiko Usui, Yuji Narita

**Affiliations:** Department of Cardiac Surgery, Nagoya University Graduate School of Medicine, 65 Tsurumai-cho, Showa-ku, Nagoya, Aichi 466-8550 Japan

**Keywords:** Vascular diseases, Stem-cell research

## Abstract

Aortic aneurysm (AA) is a vascular disorder characterized pathologically by inflammatory cell invasion and extracellular matrix (ECM) degradation. It is known that regulation of the balance between pro-inflammatory M1 macrophages (M1Ms) and anti-inflammatory M2 macrophages (M2Ms) plays a pivotal role in AA stabilization. We investigated the effects of M2M administration in an apolipoprotein E-deficient (apoE^−/−^) mouse model in which AA was induced by angiotensin II (ATII) infusion. Mice received intraperitoneal administration of 1 million M2Ms 4 weeks after ATII infusion. Compared with a control group that was administered saline, the M2M group exhibited reduced AA expansion; decreased expression levels of interleukin (IL)-1β, IL-6, tumor necrosis factor-α (TNF-α), and monocyte chemoattractant protein-1 (MCP-1); and a lower M1M/M2M ratio. Moreover, the M2M group exhibited upregulation of anti-inflammatory factors, including IL-4 and IL-10. PKH26-labeled M2Ms accounted for 6.5% of cells in the aneurysmal site and co-expressed CD206. Taken together, intraperitoneal administration of M2Ms inhibited AA expansion by reducing the inflammatory reaction via regulating the M1M/M2M ratio. This study shows that M2M administration might be useful for the treatment of AA.

## Introduction

Aortic aneurysm (AA) is a life-threatening disease, although aortic fragility and enlargement usually progress without any symptoms. Eventually, AA may result in sudden aortic rupture, which has a poor prognosis. Although significant advances have been made in surgical treatments for AA, including endovascular repair, few medical therapies are available to prevent AA growth^1,2^.

The molecular mechanisms of AA formation and progression are known to involve chronic inflammatory responses such as upregulation of interleukin (IL)-1β, IL-6, interferon (IFN)-γ, and tumor necrosis factor (TNF)-α, and degradation of extracellular matrix (ECM) components, especially elastin. These actions are caused by the upregulation of matrix metalloproteinases (MMPs), including MMP-2 and MMP-9, that are secreted from macrophages and vascular smooth muscle cells^3,4^. Infiltration of various types of inflammatory cells, including T cells, natural killer cells, lymphocytes, mast cells, and macrophages, has been observed in both human and experimental murine AA tissues, and is associated with increased levels of proinflammatory cytokines and proteases^5–10^. Macrophages in particular are thought to play a critical role in the formation and progression of AA. Recent findings have identified two major macrophage phenotypes: classically activated M1 proinflammatory macrophages (M1Ms) and alternatively activated M2 anti-inflammatory macrophages (M2Ms)^11–13^. It is known that activated M1Ms produce proinflammatory cytokines such as TNF-α, IL-1β, IL-6, and inducible nitric oxide synthase (iNOS), and chemokines such as monocyte chemotactic protein (MCP)-1. In contrast, M2Ms produce anti-inflammatory factors such as IL-10 and transforming growth factor-β (TGF-β), and reduce the expression of major histocompatibility complex class II molecules^11^. Several studies have shown that M1M and M2M subsets are associated with AA progression. Numerous M1Ms are observed at injury sites, along with the secretion of proinflammatory cytokines and chemokines. M2Ms are eventually recruited to the site, where they facilitate an anti-inflammatory response and ECM deposition^14^. Hansan et al. reported that an equal M1M/M2M ratio contributes to aneurysm stability, while an increased ratio predisposes to aneurysm rupture; as such, a balanced M1M/M2M ratio is considered to be important for maintaining aortic tissue homeostasis^15^. In our previous study, we reported that intravenous injection of bone marrow–derived mesenchymal stem cells (MSCs) resulted in transient regression of AAs by decreasing the effects of M1Ms and increasing those of M2Ms through paracrine mechanisms that resulted in anti-inflammation and tissue repair^16^. These mechanisms also confirm that equalizing the M1M/M2M ratio might contribute to AA stability. Recently, M2M-based cell therapy has been performed in clinical and experimental studies of stroke^17^, glomerulonephritis^18^, and severe cerebral palsy in children^19^. These studies showed that treatment involving M2Ms is safe and has no early adverse effects. In addition, they suggest potential applications of M2M-based cell therapy, including the modulation of inflammatory and immunomodulatory activities related to IL-1β, IL-6, TNF-α, and IFN-γ.

We hypothesized that inducing a predominance of M2Ms at the AA lesion site and regulating the M1M/M2M ratio might be a therapeutic strategy for AA treatment. In this study, we investigated the effects of M2M-based cell therapy using an apolipoprotein E-deficient (apoE^−/−^) mouse model in which AA was induced by infusion of angiotensin II (ATII).

## Results

### M2M administration inhibits AA expansion

**Figure 1:**
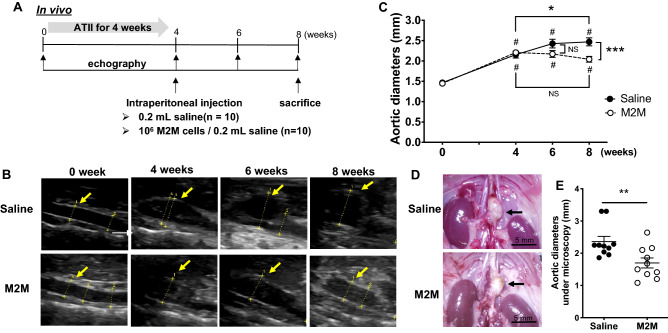
Diagram of study protocols (**A**). After ATII was administered to apoE^−/−^ mice for 4 weeks, 1 × 10^6^ M2M cells or saline was injected intraperitoneally (n = 10 mice, respectively). Mice underwent echography at 0, 4, 6 and 8 weeks, and were sacrificed at 8 weeks. (**B**) Representative long-axis echography images of the thoracoabdominal aorta. (**C**) Aortic diameters measured by echography (n = 10 mice per group). Data are means ± SEM. *p < 0.05 versus time of injection (week 4) within group. ^#^*p* < 0.05 versus just before ATII infusion (week 0) within group. ***p < 0.001 between groups at 8 weeks. Data assessed by two-way repeated measures ANOVA followed by Tukey’s multiple comparisons test. (**D**) Representative AA images obtained by microscopy (black arrows). Scale bars = 5 mm. (**E**) Aortic diameters measured by microscopy (n = 10 mice per group) Data are means ± SEM. **p < 0.01 assessed by the Mann–Whitney U test. ATII: angiotensin II, apoE^−/−^: apolipoprotein E deficient, M2M: M2 macrophage, NS: not significant.

The in vivo study protocols are shown in Fig. 1A. There were no instances of death, AA rupture, or paraplegia in any of the mice in this study. Representative echography images are shown in Fig. 1B. In the Saline group, aortic expansion was observed throughout the 8 weeks of the study, and the mean aortic diameter was significantly larger at week 8 compared with week 4 (Fig. 1C, 2.47 ± 0.10 vs. 2.16 ± 0.08 mm, *p* < 0.05). On the other hand, the mean aortic diameter in the M2M group at week 8 was not significantly larger than that at week 4, and was significantly smaller than that in the Saline group at week 8 (Fig. 1C, 2.04 ± 0.07 vs. 2.47 ± 0.10 mm, *p* < 0.001). Representative microscopic measurements obtained 8 weeks after ATII injection are shown in Fig. 1D. Aortic diameters in the M2M group were significantly smaller than those in the Saline group (Fig. 1E, 1.74 ± 0.15 vs. 2.36 ± 0.16 mm, *p* < 0.01). Comparison of echographic and microscopic aortic diameter measurements showed no significant differences (Supplementary Fig. S1).

### M2M administration suppresses elastin disruption

**Figure 2:**
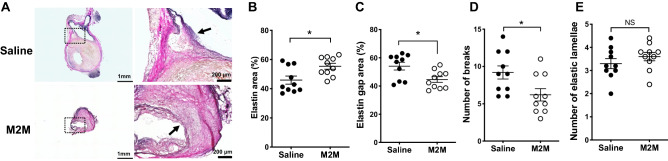
(**A**) EVG staining indicates destruction of the elastic lamellae structure (black arrows). Quantitative analysis of elastin area (**B**), elastin gap area (**C**), number of breaks (**D**), and number of elastic lamellae (**E**). Data are means ± SEM. *p < 0.05 assessed by the Mann–Whitney U test. EVG: Elastica van Gieson. NS: not significant.

Infusion of ATII for 4 weeks caused elastin disruption (Supplementary Fig. S2). At 8 weeks, the structure of the elastic lamellae was disrupted in both groups (Fig. 2A), but it was better maintained in the M2M group than in the Saline group. In the M2M group, the elastin area was better preserved from degradation compared with the Saline group (Fig. 2B, 55.4 ± 2.0% vs. 45.9 ± 2.8%, *p* < 0.05), while the medial elastin gap area was significantly decreased (Fig. 2C, 44.6 ± 2.0% vs. 54.1 ± 2.8%, *p* < 0.05). In addition, the M2M group showed significantly fewer breaks (Fig. 2D, 6.2 ± 0.8% vs. 9.2 ± 0.9%, *p* < 0.05). There was no significant difference between the two groups in the number of elastic lamellae (Fig. 2E).

### M2M administration regulates inflammatory response and MMP expression in AA

**Figure 3:**
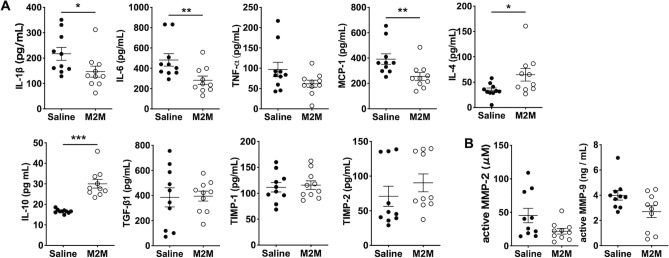
Quantitative analysis of protein expression levels in AA. (**A**) ELISA analysis of inflammatory cytokines (IL-1β, IL-6, TNF-α), a chemokine (MCP-1), anti-inflammatory cytokines (IL-4, IL-10, TGF-β1), and TIMPs (TIMP-1, TIMP-2) (n = 10 mice, respectively). Data are means ± SEM. *p < 0.05, **p < 0.01, and ***p < 0.001 assessed by the Mann–Whitney U test. (**B**) Measurement of endogenous active MMP-2 and MMP-9 (n = 10 mice per group). Data are means ± SEM.

The expression of inflammatory cytokines IL-1β and IL-6 and the chemokine MCP-1 was significantly lower in the M2M group than in the Saline group (Fig. 3A, IL-1β: 146.6 ± 26.2 vs. 216.9 ± 25.3 pg/mL, *p* < 0.05; IL-6: 282.6 ± 40.5 vs. 482.3 ± 61.2 pg/mL, *p* < 0.01; MCP-1: 255.4 ± 30.8 vs. 392 ± 41.4 pg/mL, *p* < 0.01). Among anti-inflammatory cytokines, the expression of IL-4 and IL-10 was significantly higher in the M2M group than in the Saline group (Fig. 3A, IL-4: 64.6 ± 12.5 vs. 34.1 ± 4.4 pg/mL, *p* < 0.05; IL-10: 30.1 ± 2.1 vs. 16.6 ± 0.3 pg/mL, *p* < 0.001). There was no significant difference between the two groups in the expression of TIMP-1 and TIMP-2. In addition, the M2M group showed decreased expression of endogenous active MMP-2 and MMP-9 compared with the Saline group, although the difference was not significant (Fig. 3B).

### M2M administration ameliorates the imbalance of M1Ms and M2Ms

**Figure 4:**
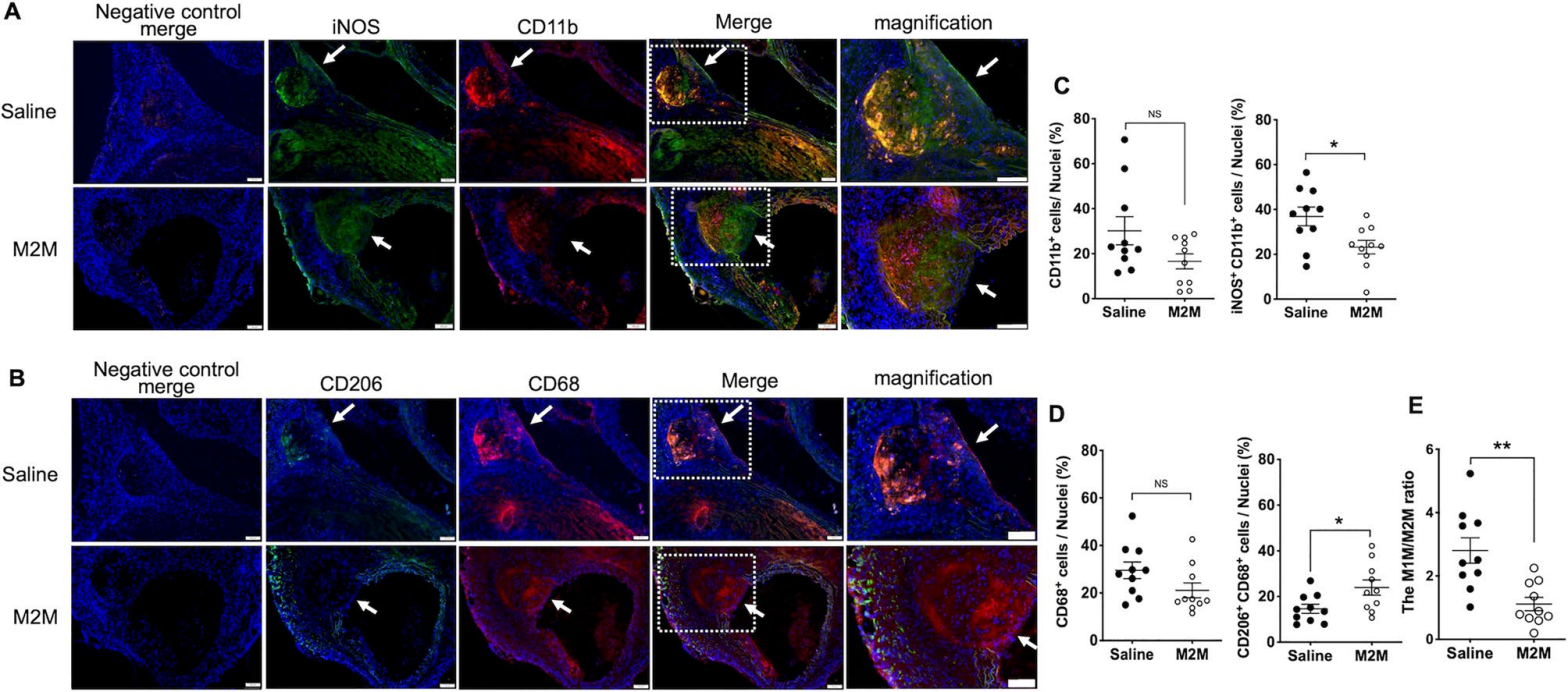
Representative images of immunofluorescence staining. (**A**) iNOS, used to detect M1Ms, is shown in green, while CD11b, used to detect macrophages, is shown in red. (**B**) CD206, used to detect M2Ms, is shown in green, while CD68, used to detect macrophages, is shown in red. Nuclei are shown in blue. Scale bars = 100 µm. (**C**) Quantitative analysis of cells positive for CD11b and iNOS. (D) Quantitative analysis of cells positive for CD68 and CD206. (**E**) The ratio of M1Ms to M2Ms. Data are means ± SEM. *p < 0.05 and **p < 0.01 assessed by the Mann–Whitney U test. M1M: M1 macrophage, M2M: M2 macrophage, NS: not significant.

Cells with an iNOS^+^CD11b^+^ double-positive phenotype were identified as M1Ms, while those with a CD206^+^CD68^+^ double-positive phenotype were identified as M2Ms (Fig. 4A,B). Abundant M1M infiltration and a lesser degree of M2M infiltration were also observed in the group that received ATII infusion for 4 weeks (Week-4 ATII group; Supplementary Fig. S3). The Saline group exhibited a significantly higher percentage of iNOS^+^CD11b^+^ cells than the M2M group (Fig. 4C, 36.9 ± 4.2% vs. 23.2 ± 3.0%, *p* < 0.05), while the M2M group exhibited a significantly higher percentage of CD206^+^CD68^+^ cells than the Saline group (Fig. 4D, 23.9 ± 3.4% vs. 14.6 ± 2.0%, *p* < 0.05). Furthermore, there was a significant difference in the percentage of CD206^+^CD68^+^ cells between the Week-4 ATII and M2M groups (Supplementary Fig. S3C). The M1M/M2M ratio was calculated by dividing the number of CD68^+^iNOS^+^ cells by the number of CD68^+^CD206^+^ cells. The M2M group showed a lower M1M/M2M ratio than the Week-4 ATII group (Supplementary Fig. S3D). In addition, The M1M/M2M ratio was significantly lower in the M2M group compared with the Saline group (Fig. 4E, 1.1 ± 0.2 vs. 2.8 ± 0.4, *p* < 0.01).

### Injected M2Ms migrate to injured AA

**Figure 5:**
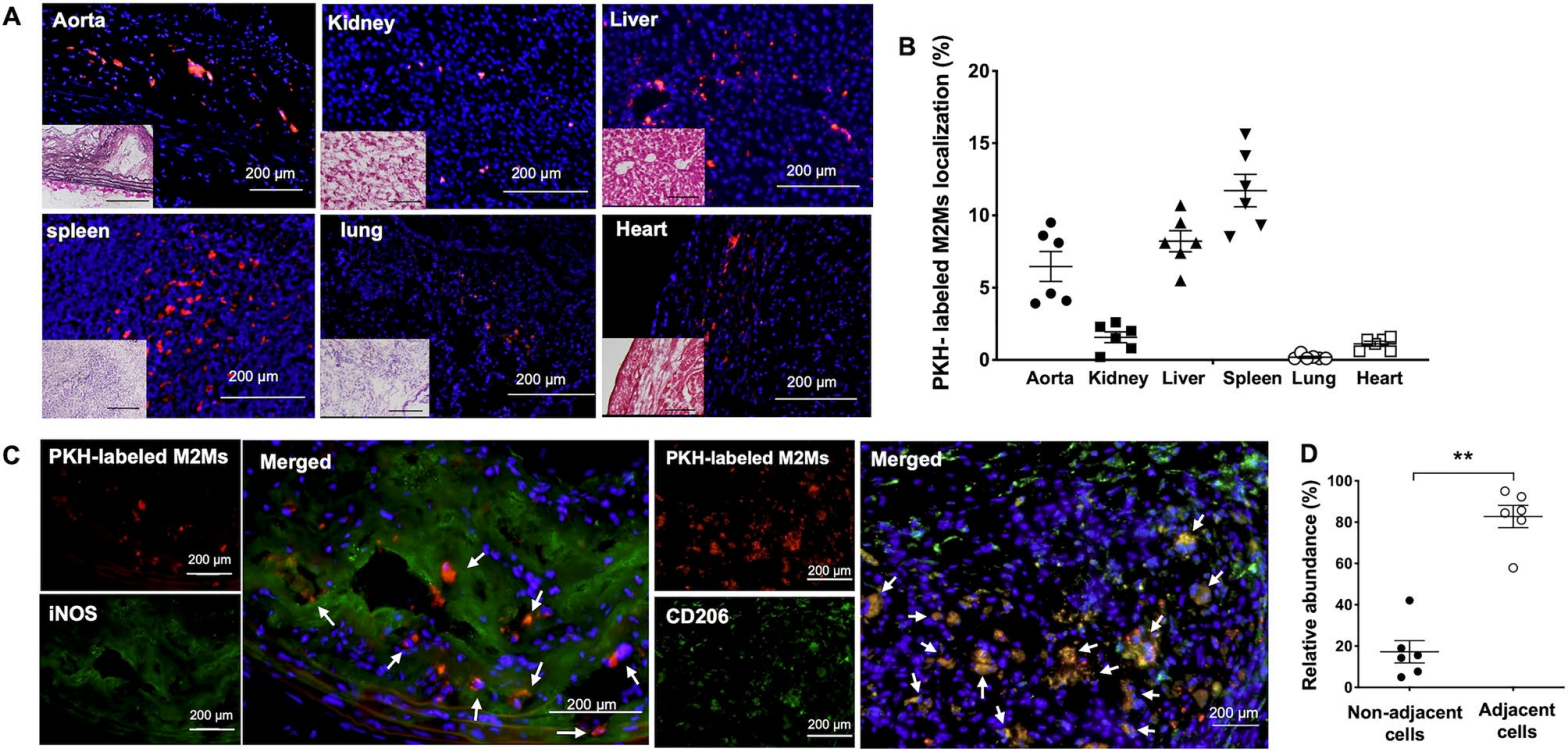
Distribution of injected M2Ms. (**A**) PKH-labeled M2Ms detected in the aorta, kidney, liver, spleen, lung, and heart. Scale bars = 200 µm. (**B**) The percentage of total cells in each tissue that were PKH-labeled M2Ms (n = 3 mice). Data are means ± SEM. (**C**) Representative images of immunofluorescence staining for iNOS and CD206 expressed on PKH-labeled M2Ms. (**D**) Relative abundance of PKH-labeled M2Ms that were localized next to iNOS. Data are means ± SEM. **p < 0.01 assessed by the Mann–Whitney U test. M2M: M2 macrophage.

Figure 5A shows PKH26-labeled M2Ms in various tissues, including the aorta, kidney, liver, spleen, lung, and heart. The percentage of total cells in each of these tissues that were PKH26-labeled M2Ms are as follows: aorta: 6.5%, kidney: 1.6%, liver: 8.2%, spleen: 11.7%, lung: 0.2%, and heart: 1.1%. (Fig. 5B). In addition, Fig. 5C shows that PKH26-labeled cells in the aneurysm site expressed CD206 but not iNOS, suggesting that injected cells maintained the M2M phenotype and did not transform into M1Ms. Approximately 82% of PKH26-labeled M2Ms were localized next to iNOS-positive cells (Fig. 5D, adjacent cells 82.7 ± 5.4% vs. non-adjacent cells 17.3 ± 5.4%, *p* < 0.01).

### M2Ms modulate mRNA expression in cocultured M1Ms and MMP amounts in cocultured AA tissue

**Figure 6:**
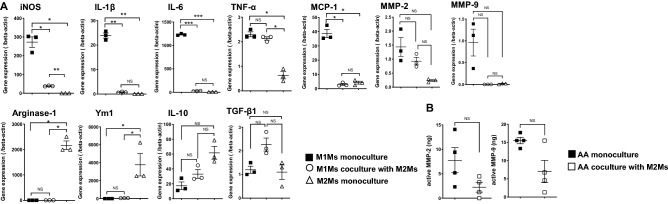
Gene expression levels in M1Ms and M2Ms, and MMP enzymatic activity levels in AA tissues. (**A**) Gene expression measured by quantitative RT-PCR. Data are means ± SEM. *p < 0.05, **p < 0.01, and ***p < 0.001 assessed by Dunnet’s T3 multiple comparisons test. (**B**) MMP-2 and MMP-9 enzymatic activity. Data are means ± SEM. M1M: M1 macrophage, M2M: M2 macrophages, AA: aortic aneurysm. NS: not significant.

We compared mRNA expression levels in M1Ms cocultured with M2Ms with those in M1M or M2M monoculture (Fig. 6A). Characteristics of macrophage phenotypes are presented in Supplementary Fig. S4. The mRNA expression of iNOS, IL-1β, IL-6, and MCP-1 was significantly lower in M1Ms cocultured with M2M than in M1M monoculture. On the other hand, M1Ms cocultured with M2Ms exhibited increased expression of IL-10 and TGF-β1 mRNA and decreased expression of MMP-2 and MMP-9 mRNA compared with M1M monoculture, although the difference was not significant. There was no significant difference in the expression of arginase-1 or Ym-1 between M1Ms cocultured with M2Ms and M1M monoculture. The amounts of endogenous active MMP-2 and MMP-9 were then measured in AA tissue. M2M coculture was associated with decreased MMP-2 and MMP-9 expression, although there was no significant difference between AA tissue cocultured with M2Ms and AA monoculture (Fig. 6B).

## Discussion

Recent studies have shown that M2Ms play a pivotal role in anti-inflammatory reactions. M2M-based cell therapy has been investigated for the treatment of diseases such as stroke, glomerulonephritis, and severe cerebral palsy, and was found to have several mechanisms of action, including modulation of inflammatory and immunomodulatory activities^17–19^. However, it is still unknown if treatments involving M2Ms are effective for aortic injury. Importantly, several papers reported the proportions of M1Ms and M2Ms in patients with atherosclerosis or AA. Atherosclerotic patients had a higher M1M/M2M ratio than non-atherosclerotic patients^20^, and the M1M/M2M ratio was positively correlated with the level of IL-1β^21^. The aortic tissue of mice with ATII-induced hypertension demonstrated a 30-fold increase in M1Ms and a five-fold increase in M2Ms compared with the aortic tissue of mice infused with saline^22^. In addition, polarization of macrophages toward the M2M phenotype in the injured aorta has been induced by various drugs and cell therapies, including eicosapentaenoic acid (EPA), docosahexaenoic acid (DHA), D-series resolvins, MSCs, and MSC-derived exosomes^16,23–25^. Hence, we hypothesized that inducing M2M accumulation in AA by M2M injection might result in an optimal M1M/M2M ratio and exert anti-inflammatory effects, thus inhibiting AA expansion. We found that intraperitoneal injection of M2Ms into AA model mice was associated with reduced inflammatory response, prevention of elastin degradation, and decreased M1M/M2M ratio. Moreover, AA tissue treated with M2Ms showed an increase in anti-inflammatory cytokines, including IL-4 and IL-10, which were confirmed to be secreted from cultured M2Ms. These results suggested that M2M administration inhibited AA expansion by improving the balance between inflammation and anti-inflammation. Notably, injected PKH26-labeled M2Ms were observed in AA tissues and expressed CD206 but not iNOS. This indicated that injected M2Ms maintained their phenotype at least throughout the observation period. Furthermore, most PKH26-labeled M2Ms were localized next to iNOS-positive cells. This suggests that the inflammatory response of M1Ms may be suppressed due to paracrine effects between M1M and M2M, a theory that was supported by gene expression changes in M1Ms cocultured with M2Ms. However, all coculture experiments were performed with an M1M/M2M ratio of 1:1. Different ratios might result in different effects.

In this study, approximately 6% of injected M2Ms were present at the AA site 4 weeks after peritoneal injection; they had maintained the M2M phenotype and did not express iNOS. A higher percentage of injected M2Ms also migrated to other organs such as the spleen. Although we carefully analyzed the site of AA injury in this study, future research should investigate systemic effects. It is unclear how long administered M2Ms retain their phenotype and their therapeutic effects, and if M1M administration might promote AA expansion by elevating the M1M/M2M ratio.

Aging is a risk factor for AAAs in humans. While current studies for ATII-induced AA have been used mice at age of 7–60 weeks^26–29^, the effect of age on the development and progression of ATII-induced AA has not been clearly shown. Apolipoprotein E-deficient mice exhibit early-stage lesions containing foam cells and smooth muscle cells at 8–10 weeks of age and advance lesions containing fibrous plaques are seen at 15–20 weeks of age^30^. The first published article by Daugherty and colleagues demonstrated that ATII-mediated atherosclerotic lesions and aneurysms occurred in 6-month-old mice^26^. Hence, we used mice ranging from 24 to 32 weeks of age in this study. A previous study showed that aged apolipoprotein E–deficient mice exhibited focal endothelial dysfunction, and impaired endothelial nitric oxide-mediated dilation occurred at a later stage of pathology^31^. These results support our finding that saline received mice exhibited gradual AA expansion despite discontinuation of ATII infusion. Meanwhile, the precise effects of M2Ms on endothelial dysfunction remain unclear and should be explored in the future.

This study has several limitations. Measurements of aortic diameters were performed using a human ultrasound system, the LOGIQ e Premium ultrasound scanner with a 10–22 MHz probe. To more accurately measure aortic diameters in mice, an advanced, ultrahigh-frequency ultrasound system for small animals should be used. Since the M2Ms used in this study are a commercially available cell line, it is necessary to confirm whether autologous monocyte-derived M2Ms can achieve the same effects. We chose to inject M2Ms intraperitoneally because intraperitoneal and intravenous administration did not result in a significant difference in AA diameters in our pilot study. Additionally, intraperitoneal injection is technically easier than intravenous administration. Regarding the number of cells administered, we injected 1 million M2Ms in 0.2 mL saline into each AA model mouse. Before doing so, we investigated whether changes in AA diameters differed according to the number of cells injected, and found that administration of 1 million, 5 million, or 10 million M2Ms in 0.2 mL saline had similar effects. However, we did not explore the effects of injecting more than 10 million cells or administering cells repeatedly. Finally, cells may transition from the M1M to M2M phenotype, and vice versa, and express genes at levels comparable to or higher than the naïve macrophage phenotypes^32,33^. However, an optimized protocol for M2Ms polarized by IL-4, IL-10, and TGF-β1 did not result in appreciable switching to M1Ms following secondary pro-inflammatory stimulation^34^. Phenotypic switching remains a matter to be discussed further. Further investigation is needed to determine whether injected M2Ms can switch to the M1M phenotype later than 4 weeks after injection.

In summary, our results demonstrated that intraperitoneal administration of M2M inhibited AA expansion by improving the balance between inflammation and anti-inflammation. This occurred by means of a decreased inflammatory response, downregulation of the M1M/M2M ratio, and an increase in anti-inflammatory cytokines, including IL-4 and IL-10, in AA tissues. This study shows that M2M administration might be useful for the treatment of AAs.

## Methods

### Macrophages

Mouse macrophage cell line J774A.1 was purchased from the Japanese Collection of Research Bioresources Cell Bank (Osaka, Japan). Cells were cultured in Dulbecco’s modified Eagle’s medium (DMEM; Sigma-Aldrich, St. Louis, MO, USA) supplemented with 10% fetal bovine serum (FBS; GIBCO, Thermo Fisher Scientific, Waltham, MA, USA). Cells were induced to differentiate into M2Ms by adding 50 ng/mL IL-4, IL-10, and TGF-β (Tonbo Biosciences, San Diego, CA, USA) to DMEM and incubating for 4 days. Cell surface antigens were identified by flow cytometry and protein expression in culture supernatants was measured by ELISA (Supplementary information).

### Animals

Experimental procedures followed the institutional guidelines for the care and use of laboratory animals and the ARRIVE guidelines. All animals were cared for in accordance with the “Guide for the Care and Use of Laboratory Animals” published by the US National Institutes of Health (Publication 85-23, National Academy Press, Washington, DC, revised in 2011). All experiments and procedures were approved by the Animal Experiment Advisory Committee of the Nagoya University School of Medicine (Protocol No. 20103). For this study, male apoE^−/−^ mice were purchased from the Jackson Laboratory (Sacramento, CA, USA).

### AA model mice

AA was induced in all 24- to 32-week-old male apoE^-/-^ mice by infusion of 1000 ng/kg/min ATII (Calbiochem, Darmstadt, Germany) for 4 weeks through subcutaneous osmotic mini-pumps (model 2004; DURECT, Cupertino, CA, USA). Implantation of mini-pumps and echography were performed under anesthesia with 1.5% isoflurane (FUJIFILM Wako Pure Chemical, Osaka, Japan) as previously described^26^.

### Intraperitoneal injection of M2Ms into AA model mice

The development of AA was confirmed by echography using a LOGIQ e Premium ultrasound scanner and a 10–22 MHz probe (GE Healthcare, Chicago, IL, USA) at 0 and 4 weeks after ATII infusion. An AA was defined as a dilated aorta with a diameter at least 1.5 times larger at 4 weeks than that at 0 weeks, following previously published guidelines^35^. We initially prepared 26 mice but excluded six of them at 4 weeks because they were non-responders with no AA expansion. After it was confirmed that aortas were dilated, 20 mice randomly received a single intraperitoneal injection of either (1) 1 × 10^6^ M2Ms (in 0.2 mL saline, n = 10 mice), or (2) saline (n = 10 mice) (Fig. 1A). M2Ms were labeled with PKH26 red fluorescent cell linker (Sigma-Aldrich) to track administered M2Ms in vivo (n = 3 mice). Four weeks after injection, the aorta was carefully exposed following sacrifice by an overdose of isoflurane, and photographed with a DP70 digital camera (Olympus, Tokyo, Japan) attached to a stereomicroscope using DP controller software (Olympus). The maximum aortic diameter at the infradiaphragm was measured using Cellsens software ver.2.3 (Olympus). Harvested aortas were evaluated by immunofluorescence staining, Elastica van Gieson staining, MMP enzymatic activity, and protein expression. Additionally, tissue samples from the aorta, heart, lung, spleen, kidney, and liver of PKH26-labeled M2M-injected mice were obtained and used for localization and immunohistochemistry.

### Echography

Two groups of mice treated with M2Ms (n = 10 mice) or saline (n = 10 mice) underwent echography using a LOGIQ e Premium ultrasound scanner and a 10–22 MHz probe (GE Healthcare) at 0, 4, 6 and 8 weeks (Fig. 1). Animals were placed in the supine position under isoflurane anesthesia, and aortic adventitial diameters were measured at the superior renal artery. The maximum diameter at 4 weeks was used as the AA diameter, and the same position was evaluated after M2M injection.

### Elastica van Gieson staining

A 2-mm-thick slice was cut from each harvested AA and embedded in OCT compound (Leica Microsystems, Buffalo Grove, IL, USA). Frozen, 10-µm-thick cross-sections were cut by a cryostat (Leica Microsystems) and stained with Weigert's resorcin–fuchsin (Muto Pure Chemicals, Tokyo, Japan), which is specific for elastic lamellae. Sections were viewed at 10× magnification and photographed (Olympus). Images were then analyzed using digital image analysis software (ImageJ v.1.41, National Institutes of Health, Bethesda, MD, USA)^36,37^ to determine the area of elastin staining as the percent area of elastin and the percent area of the medial components between the elastic lamellae (elastin gap area), both compared with the total medial tissue area, and to identify the number of elastic lamellae and the number of elastin breaks. The elastic laminae in three aortic sections per slide were assessed for the average number of elastic lamellae and the total number of breaks per elastic lamina by counting them circumferentially in all lamina. Experiments included 10 mice per group, using three consecutive aortic sections from each animal.

### Immunofluorescence staining

Aortic tissues were thoroughly washed with cold PBS to remove blood, and were then embedded using optimal cutting temperature (OCT) compound. Frozen cross-sections were fixed with 4% PFA (FUJIFILM Wako Pure Chemical) for 15 min, and blocking was performed with 10% bovine serum albumin (FUJIFILM Wako Pure Chemical). The primary antibodies used were rabbit CD11b antibody (1:200; Novus Biologicals, Centennial, CO, USA), mouse NOS2 (iNOS) antibody (1:50; Santa Cruz Biotechnology, Dallas, TX, USA), rabbit CD206 antibody (1:1000; Abcam, Cambridge, UK), and mouse CD68 antibody (1:200; Abcam). The secondary antibodies used were anti-mouse IgG (H + L), F(ab')_2_ fragment Alexa Fluor 488-conjugated antibody (1:5000; Cell Signaling Technology, Danvers, MA, USA); anti-mouse IgG (H + L), F(ab')_2_ fragment Alexa Fluor 555-conjugated antibody (1:5000; Cell Signaling Technology); anti-rabbit IgG (H + L), F(ab')_2_ fragment Alexa Fluor 488-conjugated antibody (1:5000; Cell Signaling Technology); and anti-rabbit IgG (H + L), F(ab')_2_ fragment Alexa Fluor 555-conjugated antibody (1:5000; Cell Signaling Technology). Negative control experiments used mouse IgG1 and rabbit IgG isotype control antibodies at the same concentration as the primary antibody (Cell Signaling Technology). Cell nuclei were stained with DAPI Fluoromount-G (Southern Biotech, Birmingham, AL, USA). Slides were photographed with a DP70 digital camera (Olympus) attached to a stereomicroscope using DP controller software (Olympus). Images were quantified using cellSence software ver.2.3 (Olympus). Quantification of the fluorescent signal was performed in the entire aortic wall, including the lumen, media, and adventitia.

### Measurement of protein expression and endogenous active MMP-2 and MMP-9 in AA

AA tissues were homogenized using CytoBuster™ protein extraction buffer (Novagen, Merck KGaA, Darmstadt, Germany). Lysate protein concentration was measured using a Qubit Protein Assay Kit and Qubit 2.0 fluorometer (Thermo Fisher Scientific). An equal concentration of total protein was applied to each enzyme-linked immunosorbent assay (ELISA) kit (IL-4, IL-10, IL-1β, IL-6, TGF-β1, TNF-α, TIMP-1, and MCP-1: Invitrogen, Carlsbad CA, USA; TIMP-2: Abcam), and the amount of protein was determined. Endogenous active MMP-2 and MMP-9 in aortic tissues were measured using a SensoLyte 520 MMP-2 assay kit (ANASPEC, Fremont, CA, USA) and a mouse MMP-9 activity assay kit (QuickZyme Bioscience, Leiden, the Netherlands), respectively. An equal concentration of total protein was applied to each assay kit and then detected.

### Distribution of injected M2Ms

Four weeks after injection of PKH26-labeled M2Ms, tissue samples from the aorta, heart, lung, spleen, kidney, and liver were embedded in OCT compound (Leica Microsystems) and cut using a microtome cryostat (Leica Microsystems). The frozen cross-sections (10 µm) were stained for cell nuclei using DAPI Fluoromount-G (Southern Biotech) and observed. The number of PKH26-labeled M2Ms was quantified by ImageJ and the calculation of positive percent was corrected based on the number of nuclei.

### M1Ms cocultured with M2Ms

Lipopolysaccharide-induced M1Ms were plated at 1 × 10^6^ cells per well in six-well plates and then cultured with 1 × 10^6^ M2Ms, which were cocultured using six-well plates with 0.4-µm-pore, 10-μm-thick PET membrane (4.67 cm^2^ surface area) cell culture inserts (Transwell, Corning, NY, USA). A total of 2.6 mL of a suspension of 1 × 10^6^ M1Ms was added to each plate well, and then 1.5 mL of a suspension containing 1 × 10^6^ M2Ms was plated on the insert and incubated for 48 h under the same conditions (n = 3). Monoculture (M1Ms or M2Ms alone) was used as a control (n = 3). After incubation, medium and inserts were removed and M1Ms underwent total RNA extraction.

### Total RNA extraction and quantitative real-time polymerase chain reaction

Total cellular RNA was extracted from M1Ms and M2Ms with the NucleoSpin RNA kit (Macherey–Nagel GmbH & Co. KG, Düren, Germany). Samples were normalized to 0.5 µg of total RNA. Then, cDNA was synthesized using the Takara PrimeScript RT reagent kit (Takara Bio Inc., Shiga, Japan). Quantitative real-time polymerase chain reaction (PCR) was performed based on the standard curve using Thunderbird SYBR qPCR Mix (TOYOBO, Osaka, Japan). The PCR cycling conditions were as follows: 95 °C for 1 min and then 40 cycles at 95 °C for 15 s for denaturation, followed by 60 °C for 45 s for annealing. To amplify macrophage genes, we selected primers for arginase-1, IL-1β, IL-6, IL-10, iNOS, MCP-1, MMP-2, MMP-9, TGF-β1, TNF-α, and Ym-1, along with β-actin (Sigma-Aldrich) as a control (Table 1).

**Table 1:** Primer sequences.

Gene	Accession number	Sequences of forward and reverse primers
β-actin	NM_007393	5′–AGAGGGAAATCGTGCGTGAC–3′
		5′–CAATAGTGATGACCTGGCCGT–3′
Arginase-1	NM_007482	5′–CAGCCGCCTGGAAGAGTCAG–3′
		5′–CAGATATGCAGGGAGTCACC–3′
IL-1β	NM_008361	5′–CAGGCAGGCAGTATCACTCA–3′
		5′–AGCTCATATGGGTCCGACAG–3′
IL-6	NM_031168	5′–AGTTGCCTTCTTGGGACTGA–3′
		5′–TCCACGATTTCCCAGAGAAC–3′
IL-10	NM_010548	5′–CCAGTTTTACCTGGTAGAAGTGATG–3′
		5′–TGTCTAGGTCCTGGAGTCCAGGAGACTCAA–3′
iNOS	NM_010927	5′–CCCTTCCGAAGTTTCTGGCAGCAGC–3′
		5′–GGCTGTCAGAGCCTCGTGGCTTTGG–3′
MCP-1	NM_011333	5′–CCACTCACCTGCTGCTGCTA–3′
		5′–TGGTGATCCTCTTGTAGCTC–3′
MMP2	NM_008610	5′–GTCGCCCCTAAAACAGACAA–3′
		5′–GGTCTCGATGGTGTTCTGGT–3′
MMP9	NM_013599	5′–CGTCGTGATCCCCACTTACT–3′
		5′–AACACACAGGGTTTGCCTTC–3′
TGF-β1	NM_011577	5′–TGACGTCACTGGAGTTCTACGG–3′
		5′–GGTTCATGTCATGGATGGTGC–3′
TNF-α	NM_013693	5′–TATGGCTCAGGGTCCAACTC–3′
		5′–CTCCCTTTGCAGAACTCAGG–3′
Ym-1	NM_009892	5′–GGGCATACCTTTATCCTGAG–3′
		5′–CCACTGAAGTCATCCATGTC–3′

### AA tissues cocultured with M2Ms

The aorta from the infradiaphragm to the superior mesenteric artery was harvested from AA model mice. The AA tissue was cut into round, 1-mm-thick slices. One slice of AA tissue was plated onto 24-well plates (Transwell; Corning) and then cultured with 1 × 10^6^ M2Ms that were cocultured using cell culture inserts (Transwell; Corning) (n = 4 mice). These were incubated at 37 °C in a humidified atmosphere of 5% CO_2_ in air for 7 days without media change. Monoculture (a slice of AA tissue alone) was incubated in the same manner as control (n = 4 mice). At 7 days, the incubated AA tissue was retrieved and endogenous active MMP-2 and MMP-9 were measured using a SensoLyte 520 MMP-2 assay kit (ANASPEC) and a mouse MMP-9 activity assay kit (QuickZyme Bioscience), respectively.

### Statistical analysis

Statistical significance between groups was calculated by two-way repeated measure ANOVA followed by Tukey’s multiple comparisons test, Dunnet’s T3 multiple comparisons test, Dunn’s multiple comparisons test, the Mann–Whitney U test, or the Wilcoxon matched-pairs signed rank test, as appropriate, using GraphPad Prism for Mac (Version 8; GraphPad Software, San Diego, CA, USA). All error bars are expressed as standard error of the mean (SEM). Values were considered statistically different when *P* was < 0.05.

## Supplementary Information

Supplementary Information.

## Data Availability

The datasets generated or analyzed during the current study are available from the corresponding author upon reasonable request.
