# Supplementary material for: Administration of anti-inflammatory M2 macrophages suppresses progression of angiotensin II-induced aortic aneurysm in mice
Source: Sci Rep. 2023 Jan 25;13:1380. doi: 10.1038/s41598-023-27412-x (PMC9877022; doi:10.1038/s41598-023-27412-x)
Supplement: Supplementary file 1 — Supplementary Information. [file 41598_2023_27412_MOESM1_ESM.pdf]

SUPPLEMENTARY INFORMATION

Administration of Anti-inflammatory M2 Macrophages Suppresses Progression of Angiotensin II–Induced Aortic Aneurysm in Mice

Shinichi Ashida#, Aika Yamawaki-Ogata#, Masayoshi Tokoro, Masato Mutsuga, Akihiko Usui, Yuji Narita*

Department of Cardiac Surgery, Nagoya University Graduate School of Medicine

65 Tsurumai-cho, Showa-ku, Nagoya, Aichi 466-8550, Japan

*Corresponding Author, ynarita@med.nagoya-u.ac.jp

#Equal contributors

Supplementary methods

Cell culture and macrophage differentiation

Mouse macrophage cell line J774A.1 was purchased from the Japanese Collection of Research Bioresources Cell Bank (Osaka, Japan). Cells were cultured in Dulbecco's modified Eagle's medium (DMEM; Sigma-Aldrich, St. Louis, MO, USA) supplemented with 10% fetal bovine serum (FBS; GIBCO, Thermo Fisher Scientific, Waltham, MA, USA) until nearly confluent. Naïve macrophages (M0 macrophages; M0Ms) were harvested and suspended in fresh medium, then differentiated into M1Ms or M2Ms. Inflammatory M1M differentiation was induced by 100 ng/mL LPS (Sigma-Aldrich) in culture medium for 24 h. Anti-inflammatory M2M differentiation was induced by 3 ng/mL IL-4, 10 ng/mL IL-10, and 10 ng/mL TGF-β (Tonbo Biosciences, San Diego, CA, USA) for 4 days.

Flow cytometry

To verify characterization of cultured macrophages, cells were labeled with specific surface markers and analyzed by flow cytometry. M0Ms, M1Ms, and M2Ms were collected and washed three times with PBS. One million cells were incubated at 4 °C for 2 h with the following antibodies conjugated with fluorescein isothiocyanate or phycoerythrin (eBioscience, Thermo Fisher Scientific, Waltham, MA, USA): CD11b, CD80, CD86, MHC class II, iNOS, CD206, and arginase-1 (1:100; Invitrogen, Thermo

Fisher Scientific). Labeled cells were washed and analyzed using a flow cytometer (n=3–4 mice; FACS CantoII, Becton Dickinson, Franklin Lakes, NJ, USA).

Enzyme-linked immunosorbent assay

Cell culture supernatants of M0Ms, M1Ms, and M2Ms were measured using enzyme-linked immunosorbent assay (ELISA) kits (Thermo Fisher Scientific). After macrophages differentiated, they were starved for 24 h in FBS-free DMEM. All supernatants were collected and centrifuged for 5 min at 1500 × *g*. Supernatants were then concentrated 10-fold using an AmiconUltra-4 filter with a 3-kDa molecular weight cut-off (No. UFC800324; Merck Millipore, Darmstadt, Germany). The concentration of total protein was measured using a Qubit protein assay kit (Thermo Fisher Scientific) on a Qubit 2.0 fluorometer (Thermo Fisher Scientific). For each sample, an equal volume of total protein (2.4 μg) in the wells of a 96-well plate was loaded in duplicate and detected using each ELISA kit (n=3 mice; IGF-1, IL-10: R&D Systems, Minneapolis, MN, USA; IL-1β, IL-4, IL-6, MCP-1, and TNF-α: Thermo Fisher Scientific).

Harvesting of aortas infused with angiotensin II for Elastica van Gieson and immunofluorescence staining

To evaluate elastin degradation, aortas were harvested after 4 weeks of ATII infusion (Week-4 ATII group, n=10). Frozen, 10-µm-thick cross-sections were assessed by Elastica van Gieson and immunofluorescence staining.

Supplementary Results

Aortic diameters determined by echographic and microscopic measurements

In the Saline group at 8 weeks, aortic diameters were 2.47 ± 0.09 mm by echographic measurement and 2.36 ± 0.15 mm by microscopic measurement (Supplementary Fig. S1A). In the M2M group at 8 weeks, aortic diameters were 2.04 ± 0.07 mm by echographic measurement and 1.74 ± 0.15 mm by microscopic measurement (Supplementary Fig. S1B). There was no significant difference between the two measurement methods in either group.

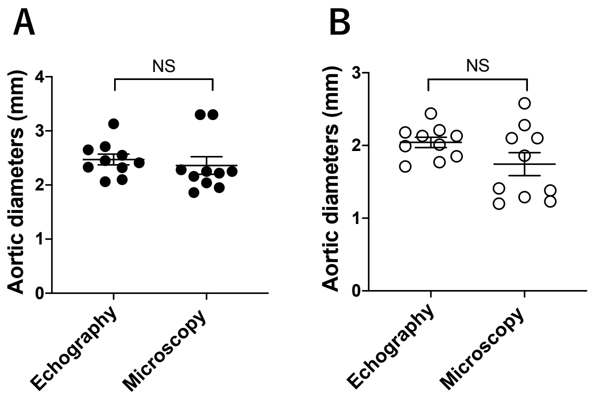

Supplementary Figure S1. Comparison of aortic diameter measurements using echography and microscopy. (A) Saline group at 8 weeks (n=10). (B) M2M group at 8

weeks (n=10). Data are means ± SEM and assessed by the Wilcoxon matched-pairs signed rank test. NS: not significant.

Elastin disruption

Infusion of ATII for 4 weeks caused elastin disruption. The Week-4 ATII group showed a smaller elastin area and larger elastin gap area compared with the M2M group (Supplementary Fig. S2A and B, Elastin area: 55.4 ± 2.0% vs 45.0 ± 2.7%, $p<0.05$; Elastin gap area: 55.0 ± 2.7% vs 44.6 ± 2.9%, $p<0.05$). In addition, the number of breaks in the Saline group was higher than in the Week-4 ATII group, but the difference was not significant (Supplementary Fig. S2C). There was no significant difference in the number of elastic lamellae between the three groups.

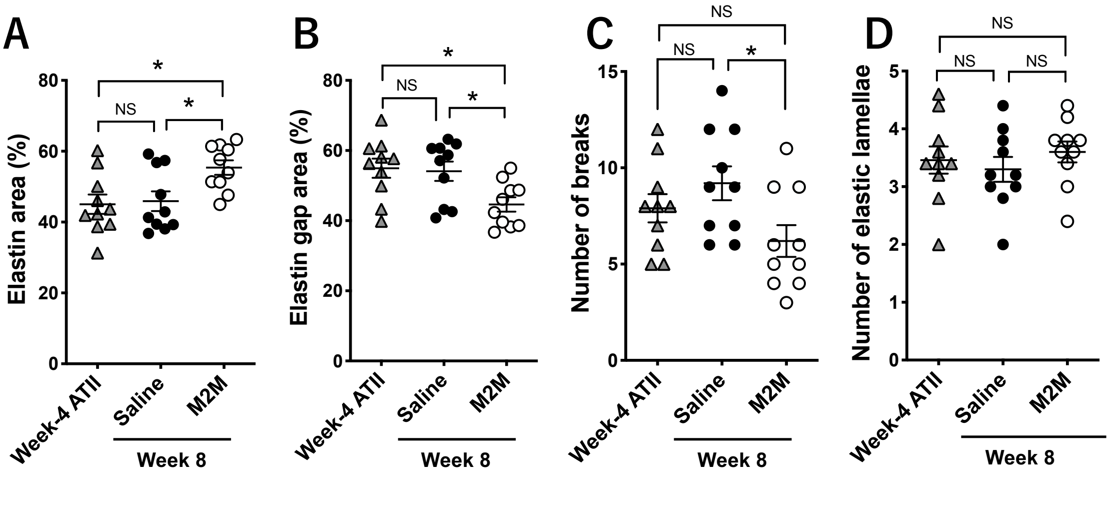

Supplementary Figure S2. Assessment of elastin disruption. Quantitative analysis of elastin area (A), elastin gap area (B), the number of breaks (C), and the number of elastic

lamellae (D). Data are means ± SEM. $^{*}p<0.05$ assessed by Dunn's multiple comparisons test. NS: not significant.

Macrophage infiltration

Abundant M1M infiltration and a lesser degree of M2M infiltration were observed in the Week-4 ATII group, which received ATII infusion for 4 weeks (Fig. S3A). Compared to the Saline group, the Week-4 ATII group showed no significant difference in the proportion of M1Ms or M2Ms, identified as $iNOS^{+}CD11b^{+}$ and $CD206^{+}CD68^{+}$ cells, respectively (Fig. S3B and C). There was a significant difference between the Week-4 ATII group and the M2M group in the percentage of $CD206^{+}CD68^{+}$ cells (Fig. S3C, 23.9 ± 3.4% vs 12.0 ± 1.3%, $p<0.05$). In addition, the M2M group exhibited a lower M1M/M2M ratio than the Week-4 ATII group (Fig. S3D, 1.1 ± 0.2 vs 2.7 ± 0.4, $p<0.01$).

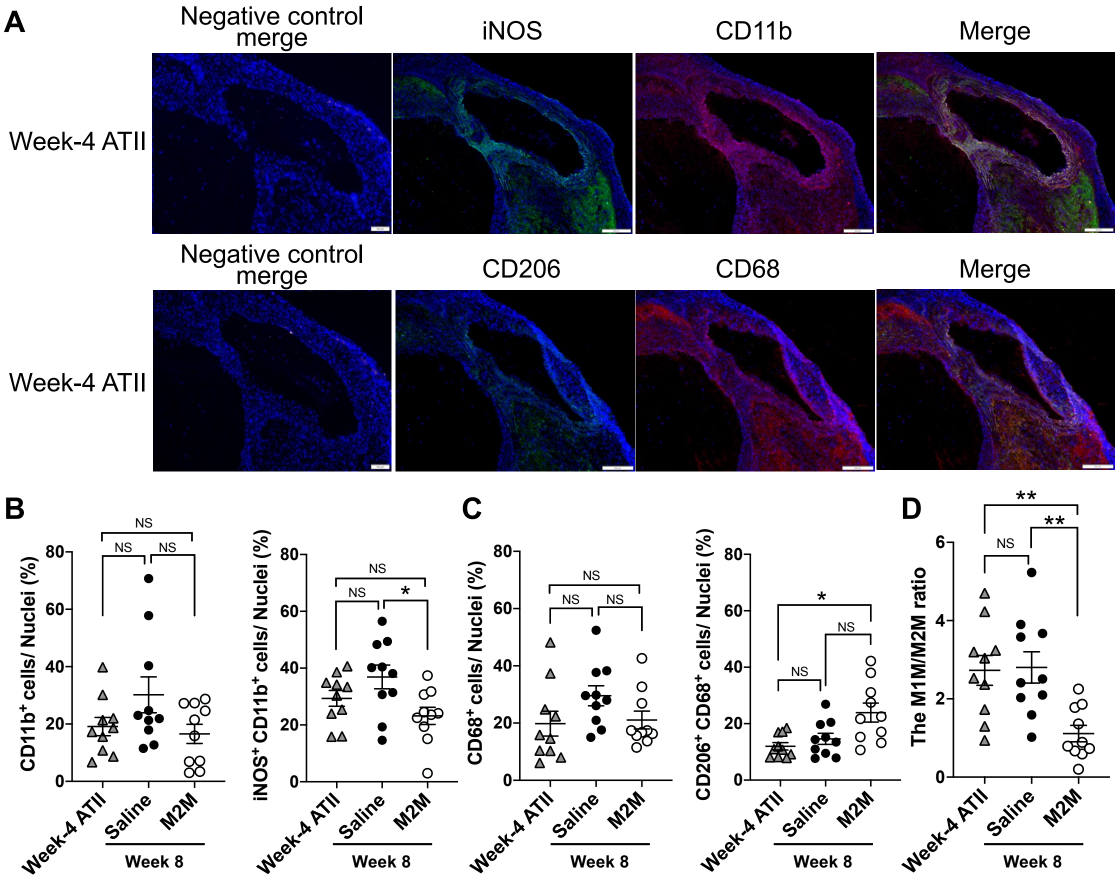

Supplementary Figure 3. Representative images of immunofluorescence staining. (A) iNOS, used to detect M1Ms, is shown in green, while CD11b, used to detect macrophages, is shown in red. CD206, used to detect M2Ms, is shown in green, while CD68, used to detect macrophages, is shown in red. Nuclei are shown in blue. Scale bars = 200 µm. (B) Quantitative analysis of cells positive for CD11b and iNOS. (C) Quantitative analysis of cells positive for CD68 and CD206. (D) The ratio of M1Ms to M2Ms. Data are means ± SEM. *p<0.05 and **p<0.01 assessed by Dunn's multiple comparisons test. M1M: M1 macrophage, M2M: M2 macrophage, NS: not significant.

Characterizations of macrophages

Cell surface expression patterns are shown in Supplemental Figure S4A. All macrophage phenotypes (M0M, M1M, and M2M) strongly expressed CD11b, a well-known macrophage marker. The CD80 and CD86 expression levels on M0Ms and M1Ms were significantly higher than those on M2Ms ($p<0.05$ for both). M1Ms expressed iNOS significantly more strongly than M2Ms ($p<0.05$). On the other hand, M2Ms expressed CD206 and arginase-1 significantly more strongly than M1Ms ($p<0.05$ for both). As shown in Supplemental Figure S4B, pro-inflammatory cytokines, including IL-1β and IL-6 were secreted significantly more strongly by M1Ms compared with M0MFs ($p<0.05$ for both). On the other hand, M2Ms secreted IL-4 and IL10 more strongly than M0Ms and M1Ms. All macrophage phenotypes strongly secreted MCP-1.

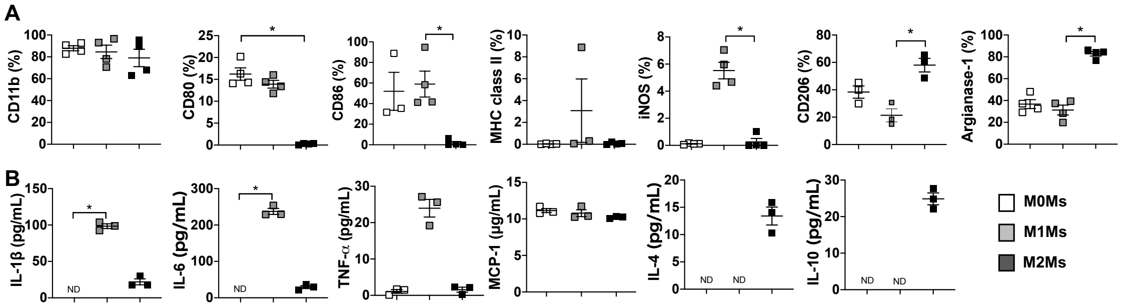

Supplementary Figure S4. Characterization of macrophages. (A) Cell surface antigens were identified by flow cytometry (n=3-4). Data are means ± SEM. *$p<0.05$ assessed by Dunn's multiple comparisons test. (B) Quantitative measurement of protein expression in the culture supernatants of M0Ms, M1Ms, and M2Ms by ELISA (n=3). Data are means

± SEM. *p<0.05 assessed by Dunn’s multiple comparisons test.
